# Residual Genetic Material in Mature Red Blood Cells

**DOI:** 10.3390/ijms262110774

**Published:** 2025-11-05

**Authors:** Georgios Dryllis, Sotirios P. Fortis, Aspasia Kouroupaki, Ioannis Tsamesidis, Vassilios Birtsas, Andreas G. Tsantes, Serena Valsami, Konstantinos Konstantopoulos, Effie G. Papageorgiou, Ilias Pessach, Anastasios G. Kriebardis

**Affiliations:** 1 Laboratory of Reliability and Quality Control in Laboratory Hematology (HemQcR), Department of Biomedical Sciences, School of Health and Caring Sciences, University of West Attica (UniWA), 12243 Athens, Greece; gdryllis@uniwa.gr (G.D.); akouroupaki@gmail.com (A.K.); vbir@uniwa.gr (V.B.); efipapag@uniwa.gr (E.G.P.); akrieb@uniwa.gr (A.G.K.); 2 Department of Biomedical Sciences, Faculty of Health Sciences, International Hellenic University, Sindos, 57400 Thessaloniki, Greece; itsamesidis@auth.gr (I.T.); iliaspessach1980@gmail.com (I.P.); 3 Laboratory of Haematology and Blood Bank Unit, Attikon University Hospital, School of Medicine, National and Kapodistrian University of Athens, 10679 Athens, Greece; 4 Hematology Laboratory and Βlood Bank, Aretaieion Hospital, National and Kapodistrian University of Athens, 11528 Athens, Greece; serenavalsami@yahoo.com; 5 Hematology Clinic, Mitera Hospital, 15123 Athens, Greece; kkonstan@med.uoa.gr

**Keywords:** red blood cells, RBC, erythropoiesis, residual genetic material, miRNAs, lncRNAs, biomarkers

## Abstract

Mature erythrocytes are traditionally regarded as anucleate cells lacking nuclear DNA. However, evidence shows they retain residual genetic material, including mitochondrial DNA (mtDNA) and RNA fragments. This review explores the role of such genetic material in cellular function, diagnostics, and erythropoiesis. A comprehensive literature review was conducted, focusing on (i) erythropoiesis, (ii) enucleation of erythroid precursors, (iii) the presence of DNA in red blood cells (RBCs), and (iv) RNA fragments such as messenger RNA (mRNA), microRNA (miRNA), and other non-coding RNAs. Mature RBCs harbor small amounts of DNA and diverse RNA species. Residual DNA can act as damage-associated molecular patterns (DAMPs), triggering immune responses when released under stress or injury. RNA fragments reflect the transcriptional activity of precursor cells and have been linked to potential diagnostic applications. Studies suggest that RBC-derived RNA signatures may serve as non-invasive biomarkers for diseases such as diabetes, cardiovascular conditions, and hematological disorders. These profiles mirror changes in erythropoiesis and provide insights into systemic pathophysiology. Residual genetic material in RBCs extends their role beyond oxygen transport. It contributes to immune modulation and may provide novel diagnostic and therapeutic opportunities, enhancing disease detection and understanding of erythropoiesis.

## 1. Introduction

Red blood cells in mammals lack a nucleus and mitochondria. Traditionally they are considered as “carrier-cells” for the oxygen and carbon dioxide gases as well as other byproducts of metabolism. During their maturation, the precursor cell of the red blood cell line undergoes chromatin condensation, movement of the nucleus to one pole of the cell, and eventually the removal of the nucleus, resulting in the creation of anucleated reticulocytes. These reticulocytes mature in the bone marrow, and then they are released into the bloodstream, where they undergo their final maturation into erythrocytes through the removal of mitochondria and other cellular organelles [1]. In many cases, after maturation in the bone marrow, characteristic structures could be observed inside the red blood cells, which are small fragments of a non-functional nucleus and contain residual nuclear DNA known as Howell–Jolly bodies (HJBs) [2,3]. Formation of HJBs is due to a defective process during erythrocyte maturation, and the majority of them contain DNA from the centromere region. Erythrocytes have been shown to regulate immune responses through the membrane receptor TLR9 (Toll-like receptor 9), which binds free mitochondrial DNA or pathogenic microbial DNA [4]. Furthermore, DNA bound to the outer membrane of erythrocytes—termed cell-surface-bound DNA (csbDNA)—has been reported, suggesting that red blood cells can act as reservoirs or carriers of extracellular nucleic acids [5]. Together, these findings indicate that erythrocytes play an active role in nucleic-acid sensing and immune modulation [6]. However, it remains unknown whether these cells have the ability to bind DNA from their environment [7,8]. In recent years, investigation of the existence of residual DNA in erythrocytes has attracted interest in research considering the possible exploitation of this specific property of these cells in understanding several diseases, including cancer [1].

## 2. Residual Genetic Material in Red Blood Cells

As already mentioned, during erythrocyte maturation, erythroid progenitor cells undergo chromatin condensation and movement of the nucleus towards one pole of the cell, ultimately resulting in the removal of the nucleus from the cell with the help of mitochondria and the creation of anucleate reticulocytes. The reticulocytes begin to mature in the bone marrow and are eventually released into the bloodstream, where their maturation is completed with the removal of mitochondria and other organelles [9]. Despite the absence of a nucleus in mature erythrocytes, these cells do not totally lack DNA and RNA. It is currently accepted that amounts of residual RNA, mainly miRNA, and DNA fragments do exist. RNA molecules are believed to play a role in the regulation of gene expression, the response to cellular stress, and intercellular communication. It is particularly interesting that the levels of miRNAs in erythrocytes may reflect the pattern of miRNAs in whole blood, serum, or plasma [10,11]. There are studies showing that the pattern of miRNA expression differs between mature erythrocytes and reticulocytes, thus supporting that miRNAs synthesized in erythrocytes constitute the main source of circulating miRNAs. This finding is of particular importance since in recent years research into diagnostic and prognostic cancer markers has focused on circulating miRNAs [12,13].

**Figure 1:**
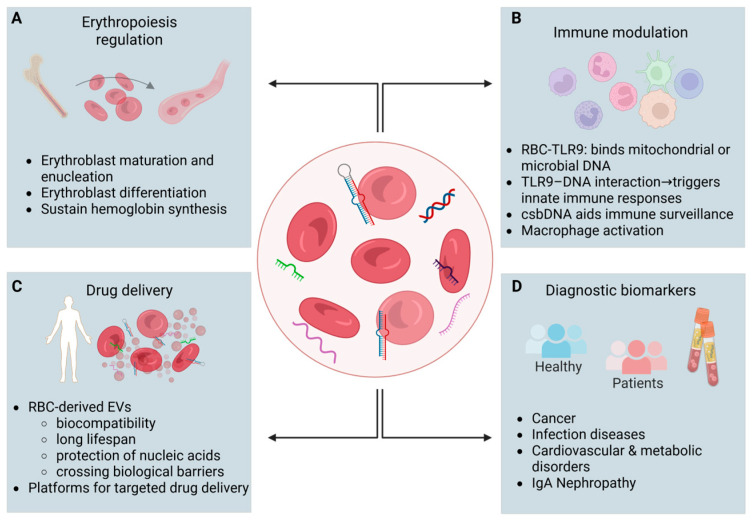
Residual genetic material in mature red blood cells (RBCs) and its potential roles. Although mature RBCs are anucleate, they retain residual RNA and DNA molecules that contribute to multiple biological and clinical functions. (**A**) Erythropoiesis regulation: Erythrocyte mRNAs and lncRNAs regulate erythroblast maturation, enucleation, and hemoglobin synthesis. (**B**) Immune modulation: RBC TLR9 binds mitochondrial or microbial DNA, triggering innate immune responses; csbDNA contributes to immune surveillance and macrophage activation. (**C**) Drug delivery: RBC-derived extracellular vesicles (EVs) show high biocompatibility, long lifespan, protection of nucleic acids, and capacity to cross biological barriers, making them natural platforms for targeted delivery. (**D**) Diagnostic biomarkers: Erythrocyte-derived RNAs serve as non-invasive biomarkers for a variety of diseases, including cancer, infectious diseases, cardiovascular and metabolic disorders, and IgA nephropathy. Created in BioRender. Fortis, S. (2025) https://BioRender.com/mku26dz (accessed on 29 October 2025).

HJBs are normally present in small numbers in peripheral blood, with their count increasing following splenectomy. An increased number of HJBs is observed following blood loss, serving as an indirect indicator of bone marrow regeneration in anemia [14]. The formation of the latter is due to errors during erythroblast development; HJBs generally contain centromeric DNA fragments, rather than euchromatic regions, originating from one or a few chromosomes depending on the defect in nuclear expulsion [3]. Overall, it has not yet been clarified whether mature erythrocytes contain DNA and what type of DNA they may contain. However, due to the property of these cells to a. circulate throughout the body, b. interact with cancer cells and promote carcinogenesis and metastasis, and c. bind DNA to the outer surface of their membrane, these cells are currently at the center of research for their potential use as carriers of targeted therapy [1] (Figure 1).

### 2.1. mRNAs

During terminal differentiation of erythroblasts, there is an accumulation of mRNA encoding hemoglobin, which constitutes 95% of total cellular mRNA [15,16]. The remaining ribosomes and mRNAs produced in the previous stages of maturation allow the continuation of translation, mainly of hemoglobin mRNAs, in reticulocytes even after enucleation [17]. In addition to hemoglobin mRNA, reticulocytes appear to contain unexpectedly diverse transcriptomes, which carry information about the expression of molecules involved in the terminal differentiation and maturation of erythroblasts [18]. However, the role and regulation of these transcripts in these final stages of reticulocyte maturation remain largely unknown, although it is known that various non-coding RNAs generally influence erythroblast differentiation [18].

The markedly increased levels of mRNAs encoding globins are due to their very long lifespan and possibly to the selective degradation of mRNA molecules that do not encode globins. In addition, many mechanisms related to the stability and degradation of mRNAs encoding α- and β-globins have been described [19]. Anucleated mature erythrocytes are unable to transcribe new mRNA molecules, and thus a gradual degradation of all RNA molecules occurs during maturation. Large-scale RNA removal occurs simultaneously with the loss of cellular structures such as mitochondria and ribosomes [19].

The predominant mRNA species in mature erythroid cells encode the globin chains of hemoglobin. In adults, these transcripts mainly correspond to α- and β-globin mRNAs, reflecting the composition of adult hemoglobin (HbA, α_2_β_2_). In contrast, during fetal development, γ-globin transcripts predominate and form fetal hemoglobin (HbF, α_2_γ_2_). Interestingly, in specific pathological conditions like β-thalassemia and sickle-cell disease, the expression of γ-globin might continue or even be reactivated. This process serves as a compensatory response that partially reinstates hemoglobin activity and attenuates the severity of the disease. This switching process of γ- to β-globin transcription is strictly controlled by chromatin modification and long non-coding RNA activity, as has been shown by recent works [20,21].

### 2.2. miRNAs: Potential Physiological Roles of Erythrocyte miRNAs

Experimental data supports that there are many miRNAs in erythrocytes and that their levels are comparable to those in nucleated cells. The development of RNA sequencing (RNA-seq) technology has allowed the identification of 359 miRNAs in mature erythrocytes, of which miR-451, miR-486, miR-144, and miR-92a have been found to be overexpressed in mature erythrocytes compared to other blood cell types [11]. The function of miRNAs in erythrocytes has been linked to erythropoiesis. For example, miR-144 and miR451 play an important role in regulating erythroblast differentiation and are notable for being found in both the human and mouse genomes [22]. miR-4732, which is overexpressed in erythrocytes, targets two key components of the TGF-β pathway (SMAD2 and SMAD4) involved in erythropoiesis, supporting the engagement of this miRNA in promoting cell proliferation during erythroblast differentiation [23,24]. Additionally, miR-486, through its interaction with GATA1, functions as a key regulator of erythropoiesis [25]. Finally, another study reports that miR-144-5p expression is affected by hypoxic environments due to elevated altitude and may play a role in erythrocyte production [26]. There is also evidence supporting a role for miRNAs in erythrocyte aging. For example, miR-196a is associated with in vitro storage-induced destruction of human erythrocytes, while miR-142 appears to play a role in controlling erythrocyte survival in mouse models. However, the mechanism by which miRNAs function in erythrocytes remains unknown [27,28,29].

### 2.3. Possible Pathological Roles of Erythrocyte miRNAs in Various Diseases

#### 2.3.1. Erythrocyte miRNAs in Infectious Diseases

**Figure 2:**
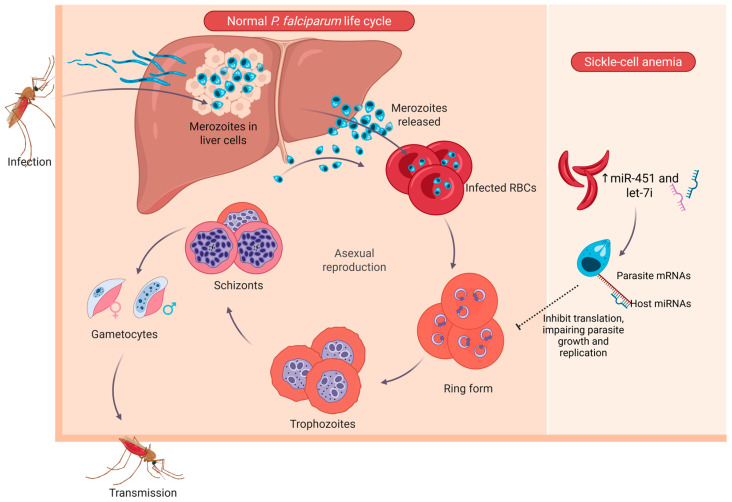
Schematic representation of the *Plasmodium falciparum* life cycle and inhibition by erythrocyte miRNAs in sickle-cell anemia. The left panel illustrates the normal asexual life cycle of *P. falciparum*, including infection of hepatocytes, release of merozoites, and intraerythrocytic development through the ring, trophozoite, and schizont stages, leading to gametocyte formation and mosquito transmission [32,33]. The right panel depicts the protective mechanism observed in sickle-cell (HbS) erythrocytes, where elevated levels of miR-451 and let-7i translocate into the parasite during the intraerythrocytic stage, bind to parasite mRNAs, and inhibit translation, thereby suppressing parasite growth and replication. Created in BioRender. Fortis, S. (2025) https://BioRender.com/mku26dz.

Studies of the role of erythrocyte miRNAs as potential etiological factors in various diseases focus mainly on infection by the Plasmodium parasite, and it appears that erythrocyte-derived miRNAs (e.g., miR-451) participate in the pathogenesis of the parasite, in the host’s defense mechanisms, and in the evolution of host polymorphisms caused by the host’s interaction with the specific parasite [30]. Previous studies have suggested that erythrocytes containing high levels of miR-451 and let-7i inhibit the proliferation of *P. falciparum*, the parasite that causes malaria and is associated with sickle cell anemia [31]. These host miRNAs can translocate from the erythrocyte cytoplasm into the parasite during the intraerythrocytic stage, where they form chimeric complexes with essential parasite mRNAs. These complexes disrupt translation of essential parasite mRNAs; they downregulate key genes involved in metabolism and replication, thereby impairing parasite development within the intraerythrocytic cycle [31] (Figure 2). This miRNA-mediated translational repression represents a unique host-derived mechanism contributing to the malaria resistance observed in sickle-cell erythrocytes.

#### 2.3.2. Erythrocyte miRNAs in Cancer

As mentioned above, nowadays it is supported that there is a correlation between miRNAs produced in erythrocytes and cancer, both in terms of its pathogenesis and diagnosis. There are studies that show that the characteristics of red blood cells are influenced by the tumor environment [34], while at the same time the problem of contamination of the plasma of cancer patients with miRNAs derived from blood cells has been highlighted [35]. Also, Han et al. reported that Ter cells, which resemble erythroblasts, are activated in the spleen and can promote carcinogenesis, suggesting that erythroblasts may be influenced by the tumor microenvironment and undergo significant changes in their physiology [36].

It appears that miRNAs derived from erythrocytes are associated with the occurrence of cancer and constitute an important source of circulating diagnostic cancer markers. It is now well established that miRNAs play a central role in the pathogenetic processes of cancer, and miRNAs, such as miR451, miR-144, miR-16, and miR-486, which are overexpressed in erythrocytes, display altered expression patterns in various types of cancer, including non-small cell lung cancer and acute leukemia [37,38,39,40]. Furthermore, despite the fact that the expression profiles of cancer-specific miRNAs in whole blood samples were initially thought to be determined by leukocytes, recent data support that other blood components, including erythrocytes and exosomes, contribute to the whole blood miRNome [41]. Wu et al. [11] conducted an interesting study in patients with colon cancer, as they analyzed the main blood miRNA markers, which are not expressed by colon tissues (miR-144-3p, miR-144-5p, miR-451a, miR-486-5p, miR-363-3p, and miR-20b-5p), in stool samples from patients and healthy donors. It was found that blood-specific miRNA-marker testing can be used as a test for the presence of blood in stool and to determine the level of heme in stool [11]. Although the aforementioned data are of particular interest, they constitute a simple indication of the contribution of erythrocyte miRNAs to cancer, and further research is certainly required to confirm the role of these specific molecules in the tumor microenvironment. The need for such studies is reinforced by the fact that in erythrocytes the translation process is inactive, and thus it is likely that they constitute a stable picture of the expression of the multitude of miRNAs they express, which regulate the onset and progression of cancer. miRNAs are also regulatory molecules in various cardiovascular and metabolic diseases. Many studies report changes in the expression of several miRNAs expressed in erythrocytes in patients suffering from these diseases [42].

#### 2.3.3. Erythrocyte miRNAs in Cardiovascular and Metabolic Regulation

**Figure 3:**
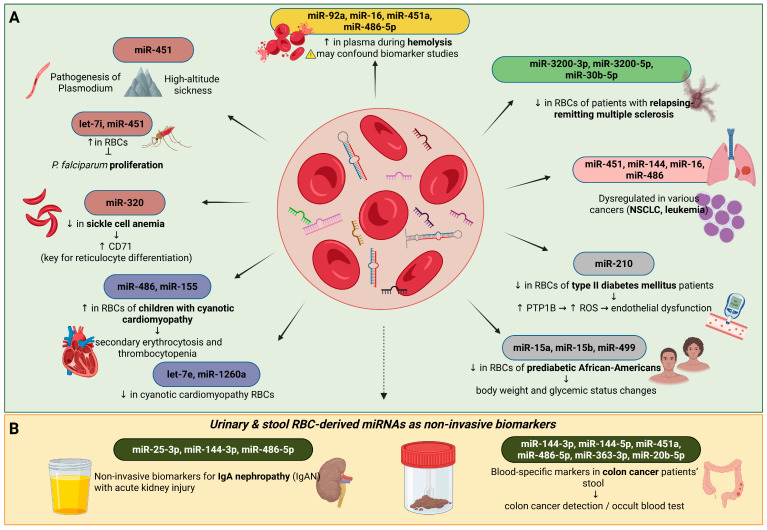
Pathological roles of erythrocyte-derived microRNAs (miRNAs). (**A**) Erythrocyte miRNAs are implicated in diverse pathological conditions and may serve as non-invasive biomarkers. In plasma, hemolysis elevates miR-92a, miR-16, miR-451a, and miR-486-5p, which may mislead interpretation; therefore, caution is required when assessing their value as biomarkers. Elevated levels of miR-451 and let-7i in red blood cells (RBCs) inhibit *P. falciparum* proliferation, while reduced miR-320 in sickle cell anemia leads to increased CD71 expression, affecting reticulocyte differentiation. Increased miR-486 and miR-155 have been observed in RBCs of children with cyanotic cardiomyopathy, associated with secondary erythrocytosis and thrombocytopenia. Conversely, let-7e and miR-1260a are reduced in cyanotic cardiomyopathy RBCs. Erythrocyte-derived miR-3200-3p, miR-3200-5p, and miR-30b-5p are reduced in relapsing-remitting multiple sclerosis. Dysregulation of erythrocyte-abundant miRNAs (miR-451, miR-144, miR-16, miR-486) has been linked to cancer, including NSCLC and leukemia. Reduced miR-15a, miR-15b, and miR-499 in prediabetic African Americans correlate with changes in body weight and glycemic status, while decreased miR-210 in type II diabetes mellitus increases PTP1B and ROS production, contributing to endothelial dysfunction. (**B**) Urine RBC-derived miR-25-3p, miR-144-3p, and miR-486-5p serve as non-invasive biomarkers for IgA nephropathy, while stool analysis of blood-specific miRNAs (miR-144-3p, miR-144-5p, miR-451a, miR-486-5p, miR-363-3p, miR-20b-5p) can detect blood/hemoglobin and may support colon cancer diagnostics. Created in BioRender. Fortis, S. (2025) https://BioRender.com/mku26dz.

The development and application of next-generation sequencing (NGS) methodology led to the observation that the levels of miR486 and miR-155 are increased in contrast to those of let-7e and miR-1260a, which are decreased in erythrocytes isolated from children suffering from cyanotic cardiomyopathy. These data support the potential contribution of erythrocyte-derived miRNAs to secondary erythrocytosis and thrombocytopenia in congenital heart disease [43]. Erythrocyte miR-15a, miR-15b, and miR-499 were downregulated in African American pre-diabetic adults, who were otherwise healthy and free of sickle cell disease, and their expression correlated with changes in body weight and glycemic status (as indicated by fasting glucose and HbA1c levels) [44]. The above studies indicate that these miRNAs can be used as future biomarkers for cardiovascular and metabolic diseases. A recent study also reports that miR-210 levels are reduced in the erythrocytes of patients with type II diabetes mellitus. In fact, this decrease leads to an increase in the levels of tyrosine phosphatase 1B, which is a target of this miRNA, thus promoting ROS production and defective vascular endothelial function [45]. These results provide evidence for the mechanistic role of erythrocyte miR-210 in endothelial dysfunction associated with type II diabetes mellitus and suggest potential new strategies for the treatment of vascular complications by restoring erythrocyte miR-210 levels (Figure 3A).

The use of erythrocyte miRNAs as biomarkers in the pathophysiology of cardiovascular and metabolic diseases requires further research. These studies should focus on investigating whether the observed changes in circulating miRNA levels are due to the pathophysiology of the disease or to erythrocyte-related events, such as hemolysis. Furthermore, assessing the value of these molecules as diagnostic, prognostic, and therapeutic tools for these diseases requires understanding a. the function of erythrocyte miRNAs, b. their contribution to cardiovascular function, and c. the mechanisms involved in the interaction between erythrocytes and the cardiovascular system.

#### 2.3.4. Erythrocyte miRNAs as Biomarkers

There are other studies that show that miRNAs derived from erythrocytes are, on the one hand, associated with abnormalities of these cells and the corresponding related diseases and, on the other hand, can be used as diagnostic biomarkers. For example, Duan et al. [46] showed that miR-25-3p, miR-144-3p, and miR-486-5p in urine samples are mainly derived from erythrocytes in urine, and this observation may serve to exploit these miRNAs as non-invasive diagnostic biomarkers for IgA nephropathy (IgAN), which is associated with acute kidney injury [46]. Furthermore, Groen et al. showed that the expression levels of erythrocyte-derived miR-3200-3p, miR-3200-5p, and miR-30b-5p are significantly reduced in relapsing-remitting multiple sclerosis [47], while a similar reduction has been observed for miR-15a, miR-15b, and miR-499 in erythrocytes of prediabetic African Americans [44]. Furthermore, erythrocyte miRNAs appear to be associated with specific pathological conditions. For example, it has been observed in patients with sickle cell anemia that underexpression of miR-320 is associated with overexpression of CD71, which plays an important role in the terminal differentiation of reticulocytes [48]. It has also been reported that the expression levels of erythrocyte miRNAs, including miR-451, are altered under high-altitude hypoxic conditions and are associated with the occurrence of acute mountain sickness (AMS) [49] and that the overexpression of miR-486-5p, miR-92a, miR-16, and miR-451a in plasma is associated with increased hemolysis [35]. Sickle cell anemia is an inherited disease, which is characterized by prolonged hemolytic anemia and vaso-occlusive episodes that can subsequently lead to additional cardiovascular complications, such as pulmonary and systemic vasculopathy, cardiac arrhythmia, or even sudden death [50].

IgA nephropathy has also been associated with the development of several vascular dysfunctions, and a recent study suggests that there is an increased risk of ischemic heart disease in these patients [51,52]. In patients with IgA nephropathy, overexpression of miR-25, miR-144, and miR-486 derived from urinary erythrocytes has been found, along with an increase in the number of extracellular vesicles isolated from the urine of patients and containing miR-144 and miR-486 (Figure 3B). In the same study, the researchers analyzed the 50 erythrocyte-derived miRNAs that showed the largest changes in their expression levels and suggested that 33 of them are potential future biomarkers for IgA nephropathy. Therefore, this study constitutes an important demonstration that miRNAs derived from either erythrocytes or extracellular vesicles can serve as non-invasive biomarkers of this disease [46].

Overall, RBC-derived miRNAs are associated with a wide range of diseases, including infections, metabolic disorders, and cancer. Their stability and abundance make them promising biomarkers. Future studies should further evaluate their potential for diagnostic, prognostic, and therapeutic applications.

### 2.4. lncRNAs

Beyond microRNAs, long non-coding RNAs (lncRNAs) have emerged as crucial regulators of erythroid development. LncRNAs are transcripts > 200 nucleotides long that do not encode for any protein. LncRNAs are categorized into six subgroups based on their position in the genome and the neighboring protein-coding genes: intergenic lncRNAs (lincRNAs), intronic overlapping lncRNAs (ilncRNAs), antisense lncRNAs (alncRNAs), enhancer lncRNAs (elncRNAs), small RNA-host lncRNAs (shlncRNAs), and pseudogene lncRNAs (plncRNAs) [53]. Compared to mRNAs, the LncRNA molecules are shorter, 2–3 exons long, less conserved across species, and characterized by lower expression levels, which also exhibit spatiotemporal specificity [54]. Their main role is regulation of gene expression that is achieved through many mechanisms.

### 2.5. LincEPS and LncRNA-Saf in Erythroid Cell Survival

The first study of lncRNAs was carried out in mouse embryonic progenitor cells (BFU-Es, CFU-Es) and Ter119+ erythroblasts at the final stage of differentiation by applying RNA-seq sequencing methodology. It was found that over 400 lncRNAs are expressed during erythropoiesis in the mouse. Among them, lincEPS (lincRNA-erythrocyte prosurvival) showed high levels of expression at the final stages of differentiation, and its inhibition led to apoptosis and significant problems during differentiation and enucleation. In contrast, enhancement of lincEPS expression had a protective effect on red blood cell progenitor cells with respect to apoptosis, indicating the potential antiapoptotic activity of lncRNA [55]. On the other hand, the lncRNA Fas-antisense 1 (Fas AS1 or Saf) is overexpressed in the final stages of human erythroblast maturation, a fact that seems to be associated with the protection of erythroblasts from Fas-induced cell death signals [56].

### 2.6. AlncRNA-EC7 and ShlncRNA-EC6 During Erythroid Differentiation

In a similar study to Hu et al. [55], Alvarez-Dominguez et al. [57] investigated the expression profile of lncRNAs at different stages of erythroblast differentiation and identified 655 differentially expressed lncRNAs; 132 of them were detected for the first time. The results of this study showed that differential expression of these molecules was closely correlated with the changes in the architecture of the histone-chromatin complex and was regulated by transcription factors that play a central role in erythroblasts, including GATA1, TAL1, and KLF1. Twelve lncRNAs displayed particularly high expression, including alncRNA-EC7 and SLC4A1. The majority of these lncRNAs are involved in erythroblast differentiation and enucleation [57]. Stage-specific expression patterns are frequently exhibited by erythroid lncRNAs, and both cis- and trans-regulatory mechanisms are employed to orchestrate transcription factor networks during terminal erythroid differentiation [58]. Furthermore, transcriptomic analyses have revealed multiple lncRNAs, such as UCA1, that modulate heme biosynthesis by stabilizing ALAS2 mRNA via their interaction with PTBP1. This establishes a direct mechanistic connection between lncRNA signaling and the metabolic regulation of erythropoiesis [21].

### 2.7. BGLT3 in the Regulation of Globin and UCA1 Gene Expression and Heme Biosynthesis

In humans, the β-globin cluster consists of five genes sequentially expressed during development. The gene encoding the lncRNA BGLT3 is located between two of these genes and specifically regulates the expression of fetal γ-globin. In erythroblasts, BGLT3 is transcribed together with the γ-globin gene and localizes to the nucleus. BGLT3 affects the chromatin structure in such a way that transcription of the γ-globin gene is possible. Given that increased levels of fetal hemoglobin can reduce the symptoms of sickle cell anemia and β-thalassemia, BGLT3 could become a therapeutic target for these diseases [58]. Shi et al. [20], applying RNA-seq at various stages of erythroblast differentiation, found that among the lncRNAs that are highly expressed during erythropoiesis, UCA1 regulates heme metabolism in erythroblasts. This lncRNA shows the highest expression levels in pre-erythroblasts, while inhibition of its expression results in the arrest of differentiation at the pre-erythroblast stage due to insufficient heme synthesis [20,21].

### 2.8. EVs (Extracellular Vesicles)

Extracellular vesicles (EVs) are heterogeneous particles surrounded by a phospholipid membrane, containing functional molecules such as RNA; they have a well-established role in intercellular communication [59]. Erythrocytes, along with platelets, are the main cells that release EVs; it has been shown that the production and release of such vesicles is important not only for the maturation of reticulocytes but also because it allows erythrocytes to remove damaged or harmful molecules, thus preventing the premature removal of these cells from circulation. The RNA content of EVs is of particular interest, especially with regard to the identification of new biomarkers [60,61]. Furthermore, it has been proposed that erythrocytes may function as a “repository” of miRNAs, which function through the release of EVs containing specific miRNAs. This hypothesis is based on experimental data comparing the miRNAs contained in erythrocytes with those in serum and exosomes. The comparison reveals a significant overlap between these materials, supporting the idea that blood contains exosomes derived from erythrocytes and that serum may contain miRNAs released by specific blood cells. The role of these RNA molecules is being intensively studied; however, data so far support that the transfer of RNA molecules mediated by EVs constitutes an effective method of transmitting messages that can modify various biological functions of the cells that receive the RNA molecules [62]. Indeed, the results of recent studies indicate that the bone marrow, lungs, liver, spleen, and kidneys may be potential targets for red blood cell miRNAs. Specifically, it has been found that i. erythrocytes contain many important miRNAs involved in the process of erythropoiesis, as shown in experimental mouse models [22]; ii. the lungs are the main organ of oxygen exchange, which implies the concentration of a large number of red blood cells and it is suspected that the miRNAs of these cells are affected in lung cancer, as well as the possible presence of platelets and exosomes [41]; and iii. the spleen, kidneys, and liver contain a large amount of intracellular vesicles—EVs, which are derived from erythrocytes, and the corresponding miRNAs may be involved in various renal diseases [38]. In addition, it has been observed that extracellular vesicles (EVs) derived from erythrocytes and containing the toxic protein asynuclein (α-syn) can cross the blood–brain barrier, thus providing another mechanism for the initiation and progression of Parkinson’s disease [63].

### 2.9. DNA

Regarding the presence of DNA in mature erythrocytes, to date there is only one study by Liang et al. [1], who experimentally confirmed the presence of nuclear and mitochondrial DNA fragments in such a subgroup. This group also found that mature erythrocytes can acquire DNA fragments from cancer cells. This observation emerged after experiments with both cancer cell lines and lung cancer patient samples. This process is believed to be mediated by the TLR9 receptor and induces the innate immune response. This potential action of erythrocytes contributes to the ability of the immune system to recognize cancer cells at early stages and present them to macrophages for destruction. Another important finding of this study was the ability to detect common lung cancer mutations in EGFR and KRAS using red blood cell DNA, a finding laying the groundwork for developing the technology for a new liquid biopsy. Finally, the same group reported that red blood cell DNA analysis can distinguish small benign from malignant lung nodules, thus offering a way for the development of new non-invasive diagnostic approaches for this type of cancer [1].

### 2.10. CircRNAs

The biogenesis of circRNAs mainly derives from exons, but they can also originate from intronic, intergenic, antisense, or untranslated regions, indicating the great diversity of their genomic sources [64,65]. They are increasingly recognized as regulators of gene expression and cell differentiation [66].

A recent comparative study showed different circRNA landscapes in fetal liver-derived erythroid progenitors versus bone marrow-derived erythroid progenitors, suggesting roles in the fetal-to-adult erythroid transition [67], and RNA sequencing work revealed an abundant repertoire of circRNAs in human erythrocytes with persistence through reticulocyte maturation [68]. The biological roles of circRNAs involve the regulation of gene expression through multiple mechanisms. They can serve as miRNA sponges, binding to miRNAs and preventing them from interacting with their target mRNAs; serve as scaffolds or decoys for RNA-binding proteins; and, in some cases, function as templates for translation of small peptides [66,69].

In erythroid cells, circRNAs regulate miRNAs involved in the processes of erythropoiesis, especially miR-451, miR-144, and miR-486, which are essential for red cell maturation, oxidative stress response, and lifespan [22,70,71]. CircRNA profiles have been shown to be altered in β-thalassemia, and networks including hsa-circRNA-100466/miR-19b-3p/SOX6 might affect globin gene expression and severity of illness [72,73]. Emerging evidence suggests that circRNAs contribute to the regulation of globin gene expression, as indicated by in silico analyses linking circRNA networks to key globin-switching regulators such as *BCL11A* and *KLF1* [67]. In stored RBCs, over 2500 known and 6000 novel circRNAs were identified, with downregulation correlating with storage duration, suggesting roles in RBC aging and storage lesions [74].

## 3. Conclusions

Red cells are a key component of blood, being responsible for the exchange of respiratory gases, for the transport of nutrients to all the cells of the body, and for the removal of useless by-products of metabolism. Mature red blood cells are unique, compared to all other cell types, due to the absence of a nucleus and other cellular organelles (e.g., mitochondria, ribosomes). For these reasons, until recently the prevailing view was that these cells (red blood cells) do not have any genetic material; this view was recently reconsidered after experimental data supporting not only the presence of DNA/RNA molecules but also various important functions.

Regarding the functional roles of the residual genetic material, it has been reported that it is involved in the various stages of erythroblast maturation and appears to be an important regulator of the erythropoiesis process, it regulates the synthesis and function of hemoglobin, and it plays a role in intercellular communication through extracellular vesicles.

Experimental data also support the involvement of this genetic material in the pathogenetic mechanisms of various diseases, including cancer. These data certainly constitute a promising ground for the utilization of residual genetic material in diagnosis and treatment. In particular, residual RNA can serve as a diagnostic biomarker in cancer, various infections, and hematological diseases. On the other hand, efforts are being made to appropriately manipulate residual RNA molecules found inside erythrocytes so as to enhance the function of these cells as oxygen transporters. In addition, EVs derived from RBCs could also be utilized for targeted drug delivery, given their physicochemical biocompatibility and ability to circulate throughout the body.

Furthermore, the study of residual genetic material in RBCs contributes to a more comprehensive understanding of erythropoiesis. It challenges the simplistic view of red cells as mere oxygen carriers, highlighting their role in immunological processes and disease. Ongoing research into the mechanisms of DNA and RNA retention, as well as their functional significance, promises to uncover new dimensions of red cell biology and its impact on human health.

The study of residual genetic material in erythrocytes is a new field of research; the results so far give rise to hopes for the utilization of these molecules in clinical practice as diagnostic biomarkers and therapeutic targets or “transport vehicles” for various substances, not only pharmaceuticals but also other important tools for future research that could lead to future practical applications.

## 4. Methods

A comprehensive literature search was conducted across the databases PubMed, Scopus, and Google Scholar, guided by the keywords “red blood cells”, “RBCs”, “erythropoiesis”, “residual genetic material”, “miRNAs”, “lncRNAs”, “biomarkers”. The strategy was subsequently refined to focus on recent studies including (i) erythropoiesis, (ii) precursor cell enucleation (expelling their nuclei to form mature RBCs), (iii) the presence of DNA in RBCs, and (iv) analyzing RNA fragments within RBCs, which provide a window into the transcriptional activity of their precursor cell, like messenger RNA (mRNA), microRNA (miRNA), and other non-coding RNA species. Only articles in English were included, published up to January 2025.

## Data Availability

Data is contained within the article.

## Abbreviations

The following abbreviations are used in this manuscript:

AlncRNA	Antisense Long non-coding RNA
AlncRNA-EC7	Erythroid-specific Long non-coding RNA
AS1	Antisense RNA 1
BFU-Es	Burst-Forming Unit–Erythroid
BGLT3	Beta-Globin Locus Transcript 3
CD71	Transferrin Receptor 1
CFU-Es	Colony-Forming Unit–Erythroid
csbDNA	Cell-surface-bound DNA
DAMPs	Damage-Associated Molecular Patterns
DNA	Deoxyribonucleic Acid
EGFR	Epidermal Growth Factor Receptor
ElncRNA	Enhancer Long non-coding RNA
GATA1	GATA-binding factor 1
IlncRNA	Intronic overlapping Long Non-Coding RNA
KLF1	Krüppel-Like Factor 1 (Erythroid-specific transcription factor)
LincRNA	Long intergenic non-coding RNA
LncRNA	Long Non-coding RNA
miRNA	Micro-RNA
mRNA	Messenger RNA
mtDNA	Mitochondrial DNA
KRAS	Kirsten Rat Sarcoma Viral Oncogene Homolog
NGS	Next-Generation Sequencing
NSCLC	Non-Small Cell Lung Cancer
PlncRNA	Pseudogene Long non-coding RNA
PTP1B	Protein Tyrosine Phosphatase 1B
RBC	Red Blood Cell
RNA	Ribonucleic Acid
ROS	Reactive Oxygen Species
ShlncRNA	Small RNA host Long non-coding RNA
ShlncRNA-EC6	Erythroid-specific long non-coding RNA
SLC4A1	Solute Carrier Family 4 Member 1
SMAD2	Mothers Against Decapentaplegic Homolog 2
SMAD4	Mothers Against Decapentaplegic Homolog 4
TAL1	T-cell Acute Lymphocytic Leukemia protein 1 (also known as SCL, transcription factor regulating erythropoiesis)
TGF-β	Transforming Growth Factor β
TLR9	Toll-Like Receptor 9
UCA1	Urothelial Cancer Associated 1 (lncRNA, regulator of heme metabolism in erythroblasts)
